# Structural basis for the broad substrate specificity of the human tyrosylprotein sulfotransferase-1

**DOI:** 10.1038/s41598-017-07141-8

**Published:** 2017-08-18

**Authors:** Shinnosuke Tanaka, Toshiaki Nishiyori, Hidetaka Kojo, Reo Otsubo, Moe Tsuruta, Katsuhisa Kurogi, Ming-Cheh Liu, Masahito Suiko, Yoichi Sakakibara, Yoshimitsu Kakuta

**Affiliations:** 1 Department of Bioscience and Biotechnology, Faculty of Agriculture, Kyushu University, Hakozaki 6-10-1, Fukuoka, 812-8581 Japan; 2 Graduate School of Systems Life Sciences, Kyushu University, Hakozaki 6-10-1, Fukuoka, 812-8581 Japan; 3 Food Research Branch, Department of Biochemistry and Applied Biosciences, Faculty of Agriculture, University of Miyazaki, Miyazaki, 889-2192 Japan; 4 Department of Pharmacology, College of Pharmacy, University of Toledo Health Science Campus, Toledo Ohio, 43614 USA

## Abstract

Tyrosylprotein sulfotransferases (TPSTs) are enzymes that catalyze post-translational tyrosine sulfation of proteins. In humans, there are only two TPST isoforms, designated TPST1 and TPST2. In a previous study, we reported the crystal structure of TPST2, which revealed the catalytic mechanism of the tyrosine sulfation reaction. However, detailed molecular mechanisms underlying how TPSTs catalyse a variety of substrate proteins with different efficiencies and how TPSTs catalyze the sulfation of multiple tyrosine residues in a substrate protein remain unresolved. Here, we report two crystal structures of the human TPST1 complexed with two substrate peptides that are catalysed by human TPST1 with significantly different efficiencies. The distinct binding modes found in the two complexes provide insight into the sulfation mechanism for these substrates. The present study provides valuable information describing the molecular mechanism of post-translational protein modifications catalysed by TPSTs.

## Introduction

Tyrosine sulfation, first discovered in bovine fibrinogen, is a major post-translational modification^1^ that occurs widely among proteins in multicellular eukaryotic organisms^2, 3^. Although the functional importance of protein tyrosine sulfation is not fully resolved, it has been implicated in altering biological activities of proteins, proteolytic processing of bioactive peptides^4^, influencing the half-life of proteins in circulation^5^, and modulates extracellular protein-protein interactions, as observed for inflammatory leukocyte adhesion^6, 7^. The recent discovery of tyrosine sulfation of chemokine receptors suggests an even broader role in inflammatory responses^8, 9^. For the chemokine receptor CCR5, sulfated tyrosine residues located in the receptors N-terminal extracellular region have been shown to be crucial in mediating HIV binding/infection^10–13^. Furthermore, previous studies have demonstrated the involvement of tyrosine sulfation in the activation of complement component C4^14^, maturation of progastrin to gastrin^15^ and binding of P-selectin glycoprotein ligand-1 (PSGL-1) to P-selectin^6^.

**Figure 1:**
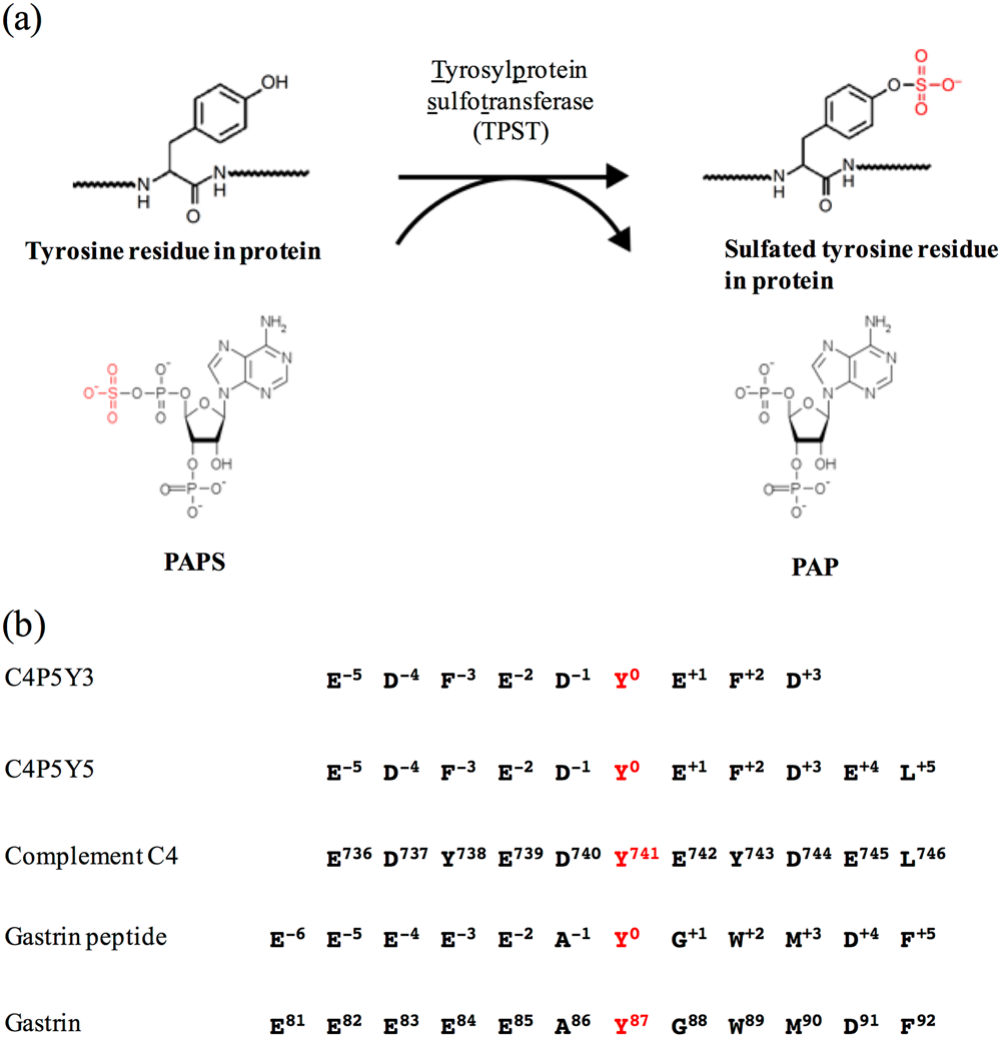
Post-translational tyrosine sulfation reaction catalysed by TPST. (**a**) The tyrosine residue of a substrate protein is sulfated by TPST using the donor substrate, PAPS. (**b**) The amino-acid sequences of the substrate peptides used in this study. C4P5Y3 corresponds to an nine-amino-acid region encompassing the tyrosine sulfation site Y741 of human complement C4. Two Tyr residues (Y738 and Y743) in the original sequences have been mutated to Phe and only one Tyr (Y741) residue exists in the peptide. C4P5Y5 corresponds to an eleven-amino-acid region encompassing the tyrosine sulfation site Y741 of human complement C4. Two Tyr residues (Y738 and Y743) in the original sequences have been mutated to Phe and only one Tyr (Y741) residue exists in the peptide. The gastrin peptide corresponds to a twelve-amino-acid region encompassing the tyrosine sulfation site Y87 of human gastrin.

Tyrosylprotein sulfotransferases (TPSTs) are enzymes that catalyse the transfer of a sulfonate moiety from 3′-phosphoadenosine-5′-phosphosulfate (PAPS) to the hydroxyl group of a tyrosine residue to form a tyrosine *O*-sulfate ester and 3′-phosphoadenosine-5′-phosphate (PAP) (Fig. 1a). TPSTs are integral membrane glycoproteins located in the *trans*-Golgi network^2^. In humans, two TPST isoforms, termed TPST1 and TPST2, have been identified^16–18^. TPST1 and TPST2 consist of 370 and 377 amino acid residues, respectively, and share 64% amino acid sequence identity (Supplementary Fig. S1). Several previous studies about the comparison of substrate specificity between the two isoforms^18–24^ show slightly different substrate specificities or basically same specificities. While TPST1 and TPST2 display levels of expression among different organs/tissues, they do share some common enzymatic characteristics^19^. Both TPST1 and TPST2 have the type II transmembrane topology with a short N-terminal cytoplasmic domain, a single 17-residue transmembrane (TM) domain and a putative stem region of ~40-residues, followed by a luminally-oriented catalytic domain^17, 18^. The only two sulfotransferases (human TPST1 and TPST2) function by sulfating a variety of substrate proteins with different efficiencies^19, 20^.

Multiple tyrosine residues in a protein substrate can be sulfated by human TPSTs. For example, a study demonstrated a sequential tyrosine sulfation of CXC chemokine receptor 4 (CXCR4) by human TPSTs^21^. In a second example, three tyrosine residues (Tyr 738, Tyr741, and Tyr743) in complement C4 are sulfated by TPSTs^25^. In the complement activation cascade, C4 is cleaved into fragments C4a and C4b by MASP-2 protease^14^. An acidic residue and three sulfotyrosine residues in the C4 α-chain are recognised by the positively charged surface of MASP-2 through electrostatic interactions. Thus, sulfation of tyrosine residues is thought to play important roles in various functional modifications that are controlled by TPSTs.

We recently solved the crystal structure of human TPST2 complexed with PAP and a substrate peptide (designated C4P5Y3, Fig. 1) derived from complement C4 at 1.9 Å resolution^26^ (Supplementary Fig. S2). The structure contained only the lumenal portion of the enzyme, and lacked the N-terminus and the TM domain. The structure revealed the molecular basis for the catalysis being based on an S_N_2-like in-line displacement mechanism. Furthermore, human TPST2 recognises the C4P5Y3 peptide in a deep cleft via a short parallel β-sheet type interaction and the bound C4P5Y3 forms an L-shaped structure. Based on the structural features and bioinfomatic analysis of substrates, the vicinity of the acceptor tyrosine residue in substrate proteins adopts an intrinsically unfolded conformation in order to facilitate access to the deep active site cleft of human TPST2^26, 27^. The mechanism underlying how human TPSTs may accommodate a variety of substrate proteins with different efficiencies, however, how human TPSTs recognise multiple tyrosine residues in substrate proteins remains unclear.

Here we report two crystal structures of the human TPST1 complexed with PAP and a complement C4 peptide (C4P5Y5) or a gastrin peptide (Fig. 1b). The structure also contained only lumenal portion of the enzyme, without the N-terminus and the TM domain. Structural information derived from the current study reveals the mechanism underlying how human TPSTs recognise a variety of substrate proteins with different efficiencies and how human TPSTs recognise multiple tyrosine residues in a substrate protein.

## Results

### Enzymatic characterisation

The sulfotransfer efficiencies of human TPST1 toward two substrate peptides, C4P5Y5 and the gastrin peptide, were quantitatively evaluated (Table 1). Kinetic analysis showed that human TPST1 displayed *K*_*m*_ values of 7.67 µM and 6.52 × 10^2^ µM for C4P5Y5 and the gastrin peptide, respectively. In our previous study, crystal structures of human TPST2 and kinetic constants indicated that Asp residue at the −1 position and the acidic residue at the +1 position are major determinants of the *K*_m_ values^26^. In line with this notion, C4P5Y5, which contains these two acidic residues, showed a lower *K*_m_ value than the gastrin peptide, which does not carry these two acidic residues. Thus, the gastrin peptide is considered to belong to a group, which includes CCR3 and CCR5, with high *K*_m_ values. Furthermore, the resulting kinetic efficiencies (*k*_cat_/*K*_m_) of human TPST1 for the gastrin peptide were reduced 633-fold in comparison to that for C4P5Y5. These results indicated that human TPST1 sulfates more efficiently C4P5Y5 than the gastrin peptide.

**Table 1:** Kinetic parameters of human TPST1 with substrate peptides.

Substrate	*K*_m_ (µM)	*k*_cat_ (min^−1^)	*k*_cat_/*K*_m_ (min^−1^µM^−1^)
C4P5Y5 peptide	7.67 ± 1.7	4.90 × 10^−2^ ± 2.4 × 10^−3^	6.4 × 10^−3^
Gastrin peptide	6.52 × 10^2^ ± 4.1 × 10	6.57 × 10^−3^ ± 4.5 × 10^−4^	1.0 × 10^−5^

### Overall structures of two human TPST1 crystal structures

**Figure 2:**
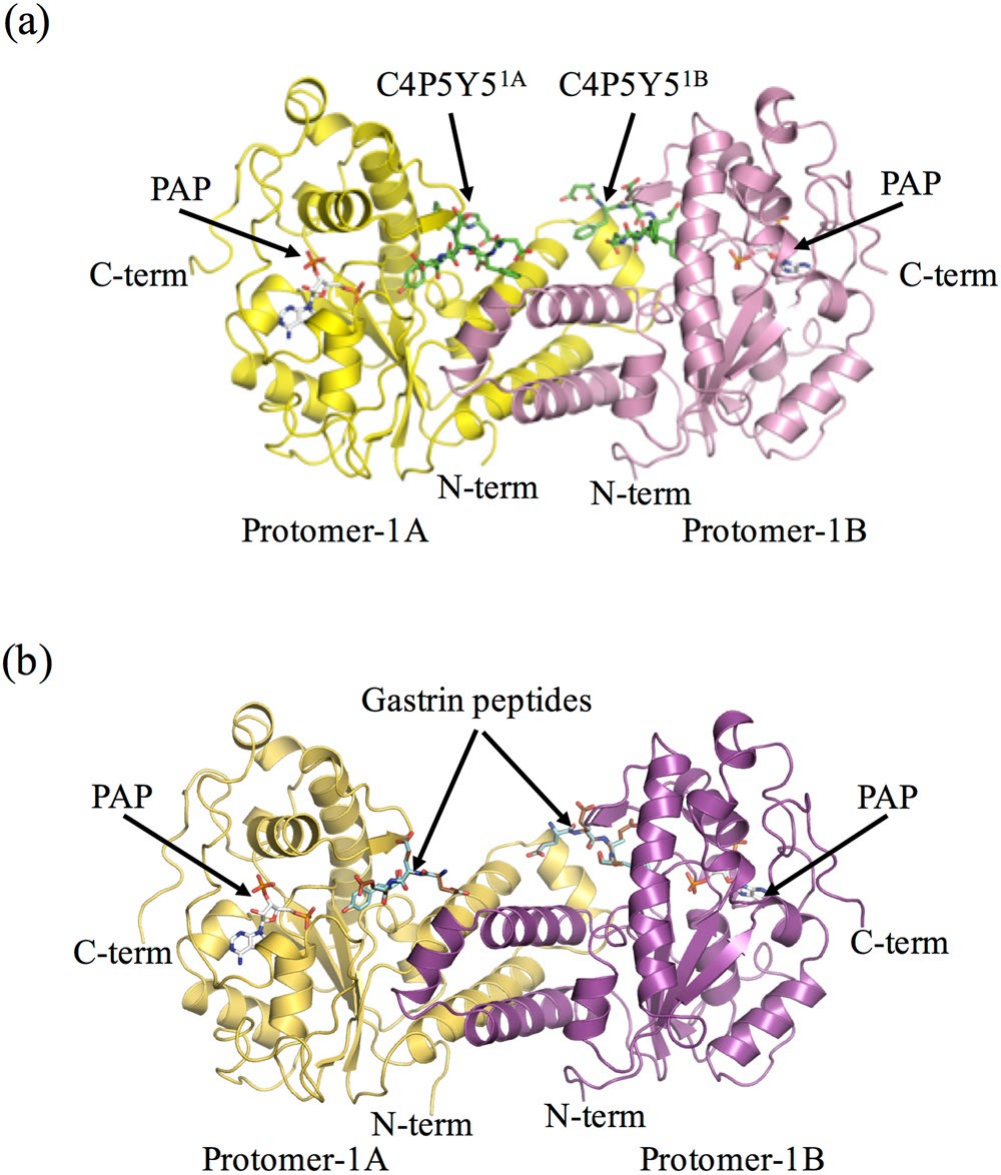
Overall structures of recombinant human TPST1. (**a**) Ribbon diagram of the structure of human TPST1-PAP-C4P5Y5. Protomer-1A and protomer-1B are yellow and pink, respectively. C4P5Y5 is green. PAP is white. (**b**) Ribbon diagram of the structure of human TPST1-PAP-gastrin peptide. Protomer-1A and protomer-1B are yellow-orange and purple, respectively. Productive form and nonproductive form of the gastrin peptide are light blue and brown. PAP is white. The dimeric complex has two active sites and binds two C4P5Y5s. To distinguish between two bound C4P5Y5s in human TPST1-PAP-C4P5Y5, the peptide sulfated by protomer-1A and protomer-1B are referred to as C4P5Y5^1A^ and C4P5Y5^1B^, respectively. The structures were prepared by Pymol (http://pymol.sourceforge,net).

To investigate the structural basis for the different efficiencies of human TPST1 toward different substrates, we have determined the crystal structures of two ternary complexes of human TPST1. Crystal structures of a core domain of human TPST1 complexed with PAP and C4P5Y5, or the gastrin peptide were solved at 1.6 Å and 2.3 Å resolutions, respectively (Fig. 2a,b). The data collection and refinement statistics are summarized in Table 2. The asymmetric unit of human TPST1-PAP-C4P5Y5 contains two human TPST1 molecules, which assemble to form a dimer. In contrast, the asymmetric unit of TPST1-PAP-gastrin peptide contains four human TPST1 molecules, which assemble to form two dimers. The r.m.s.d. (root mean square deviation) values for alignment of the protomers (using 270 Cαs) are between 0.236 Å and 0.369 Å. Therefore, all protomers of human TPST1 are almost structurally identical (Fig. 2a,b). While a part of the N-terminal and C-terminal region observed in the crystal structure of human TPST2 was not observed in the crystal structure of human TPST1, the majority of the secondary structure of human TPST1 is very similar to that of human TPST2 (Supplementary Figs S1 and S3). The human TPST1 catalytic domain comprises a single α/β motif with a five-stranded parallel β-sheet, flanked on both sides by α helices, and this structure is consistent with human TPST2 structure (Supplementary Figs S2 and S3). The 5′-phosphosulfate-binding (5′-PSB) motif, which is contained within a strand-loop-helix consisting of β3 and α1, is central to this structural motif. Moreover, β6 and α7 are also key elements that include the 3′-phosphate-binding (3′PB) motif. The 5′-PSB and 3′-PB motifs are conserved among all members of the sulfotransferase family. Structural comparison of human TPST1-PAP-C4P5Y5 with human TPST2-PAP-C4P5Y3 yields an r.m.s.d. of only 0.814 Å for 272 Cαs, suggesting that the two structures are very similar (Supplementary Fig. S3). Furthermore, the positions of the catalytic residues R79, E100, K159 and S286 in human TPST1 are almost identical to those of the catalytic residues R78, E99, K158 and S285 in human TPST2, suggesting that the catalytic mechanism of human TPST1 is basically same to that of human TPST2 (Supplementary Figs S4 and S5). These results are in support of similar enzymatic characteristics found for the two human TPSTs.

**Table 2:** Data collection and refinement statistics.

Data collection	TPST1-PAP-C4P5Y5	TPST1-PAP-gastrin peptide
Space group	*P*2_1_2_1_2	*P*1
Unit cell parameters	*a* = 78.6 Å	*a* = 51.7 Å
	*b* = 156.0 Å	*b* = 93.3 Å
	*c* = 48.9 Å	*c* = 93.0 Å
		α = 90.6°
		β = 93.4°
		γ = 103.4°
Wavelength (Å)	1.3000	1.0000
Resolution range (Å)	50.0–1.60	50.0–2.33
No. of reflections (Observed/Unique)	457695/78540	123218/71093
Redundancy	5.8 (2.5)	1.9 (1.7)
*R*_*sym*_ (overall/outer shell)	0.084 (0.761)	0.155 (0.982)
*I/σ* (*I*)	20.1 (0.91)	8.33 (0.94)
*CC*_*2/1*_	(0.55)	(0.32)
Completeness (%)	98.0 (81.8)	97.5 (90.1)
Refinement statistic		
Resolution range (Å)	31.3–1.60	50.0–2.33
No. of reflections		
Working set/Test set	74483/3941	67464/3560
Completeness (%)	97.68	95.92
*R*_*cryst*_ (%)/*R*_*free*_ (%)	13.3/17.8	24.10/26.94
Root mean square deviation		
Bond length (Å)	0.011	0.007
Bond angles (°)	1.516	1.175
Average B-factor (Å^2^)/No. of atoms		
Protein	18.85/2402	46.36/8812
Ligand (PAP)	15.00/54	38.54/108
Ligand (Peptides)	30.83/76	58.18/268
Mg^2+^	16.46/2	46.24/4
Zn^2+^	20.84/1	
Water	33.12/571	38.37/144
Glycerol	21.99/6	
Ramachandran analysis (%)		
Favored	97.76	97.98
Allowed	1.79	1.56
Outlier	0.45	0.46
PDB ID	5WRI	5WRJ

Each structure was determined from a single crystal. Values in parentheses are for highest-resolution shell.

### Dimer interface

Consistent with the crystal structure of human TPST2, human TPST1 forms a homodimer (with the two subunits designated, respectively, as protomer 1 A and 1B), and is mediated by three consecutive α-helices (α2, α3 and α4) (Fig. 2, Supplementary Figs S2 and S6). In addition, structural comparison of the three α-helices (α2, α3 and α4) of human TPST1-PAP-C4P5Y5 with that of human TPST2-PAP-C4P5Y3 shows an r.m.s.d. of 0.349 Å for 79 Cαs. The sequences of three consecutive α-helices (α2, α3 and α4) in human TPST1 are homologous to those of human TPST2, except for two residues (Ser117 and Ser136). Furthermore, the dimer interface of human TPST1 shows 1471 Å^2^ of buried surface area of each protomer, whereas that of human TPST2 shows 1531 Å^2^ of buried surface area of each protomer. These results suggest that both human TPST1 and TPST2 contain a similar dimer interface (Supplementary Fig. S6). A recent report demonstrated that human TPST1 and TPST2 can form heterodimers *in vivo* ^28^. Our results showed the consistency of the dimer interface of human TPSTs, supporting the possibility of heterodimerization of human TPST1 and TPST2. In short, dimerization of human TPST1 indicated that the dimer formation completes the active site of the enzyme and produces a widely positive charged surface.

### Observation of peptides in the two crystal structures

**Figure 3:**
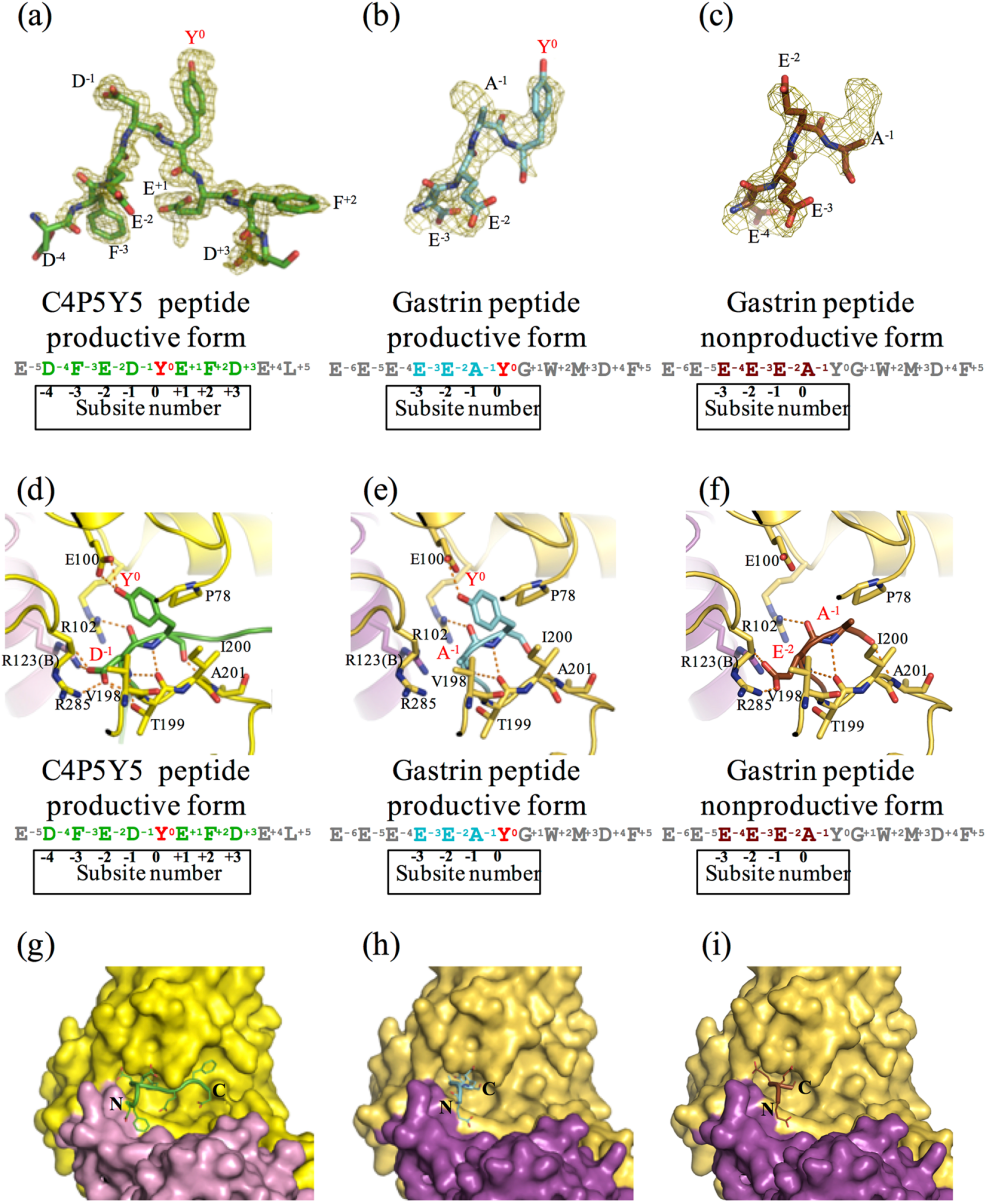
Three substrate peptides in the active site of human TPST1. (**a–c**) The electron density maps showing the image of C4P5Y5 and the gastrin peptide (simulated annealed omit Fo–Fc maps contoured at 2.5 σ and 1.7 σ, respectively). The Fo–Fc maps are drawn in olive. (**a**) Fo–Fc map of C4P5Y5. (**b**) Fo–Fc map of the gastrin peptide productive form. (**c**) Fo–Fc map of the gastrin peptide nonproductive form. (**d–f**) Interaction of Y^0^ and the residue at the subsite -1 in substrate peptides with human TPST1. Hydrogen bonds are depicted as orange dotted lines. The main chain and side chain of Y^0^ and the residue at the subsite -1 in the substrate peptide are shown. Only the main chain of the other residues in the substrate peptide is shown. Each amino acid sequence is respectively shown below. (**d**) Human TPST1-PAP-C4P5Y5. (**e**) The human TPST1-PAP-gastrin peptide productive form. (f) The human TPST1-PAP-gastrin peptide nonproductive form. (**g–i**) Close-up views of the binding site of C4P5Y5 and the gastrin peptide by surface representation. (**g**) Human TPST1-PAP-C4P5Y5. (**h**) The human TPST1-PAP-gastrin peptide productive form. (**i**) The human TPST1-PAP-gastrin peptide nonproductive form.

**Figure 4:**
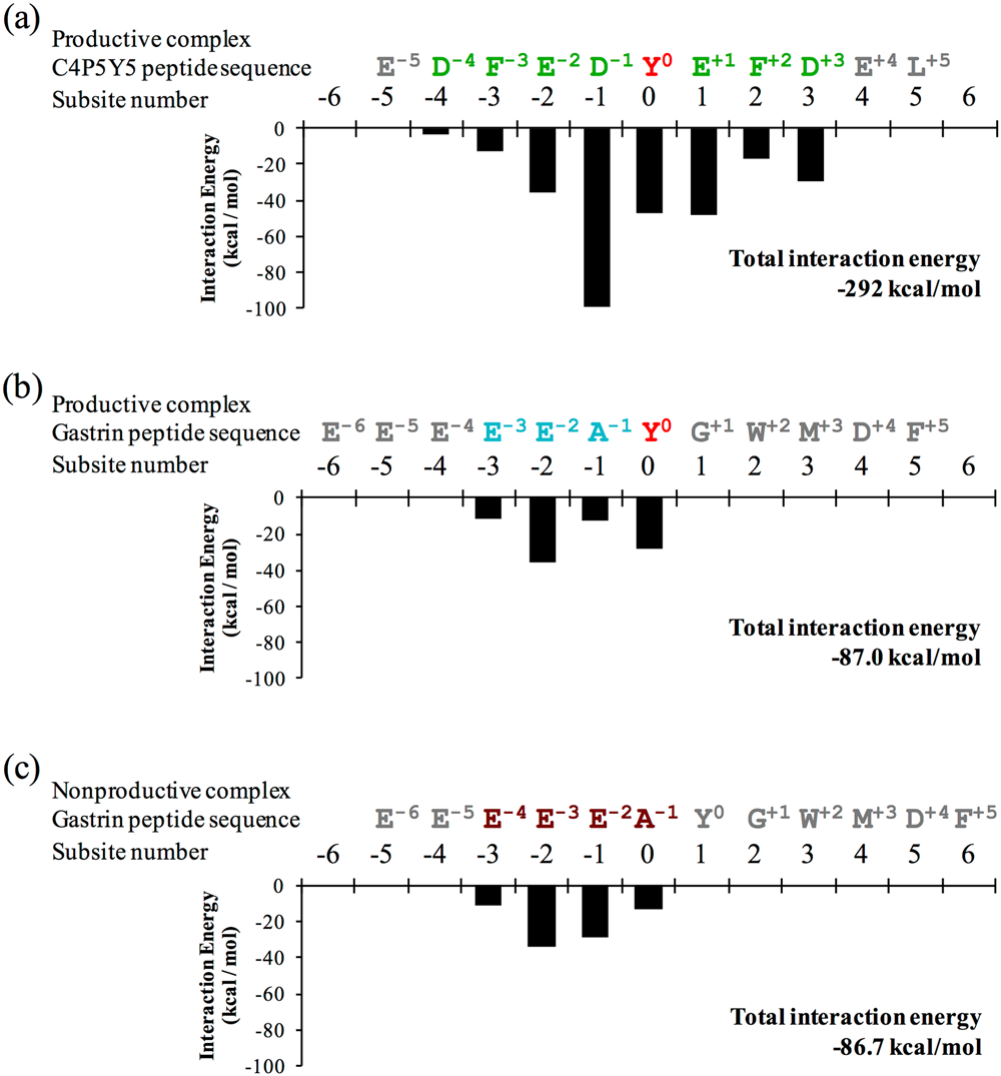
Interaction energy of human TPST1 with substrate peptides. The black bar graph illustrates the interaction energy of each amino acid in human TPST1 with substrate peptides. The total interaction energy of human TPST1 with respective substrate peptides is presented in the bottom right corner of each bar graph. The interaction energy between human TPST1 and an amino acid in each substrate peptide is shown. The amino acid sequences of the substrate peptides and subsite numbering are shown at the top of the each bar graph.

**Figure 5:**
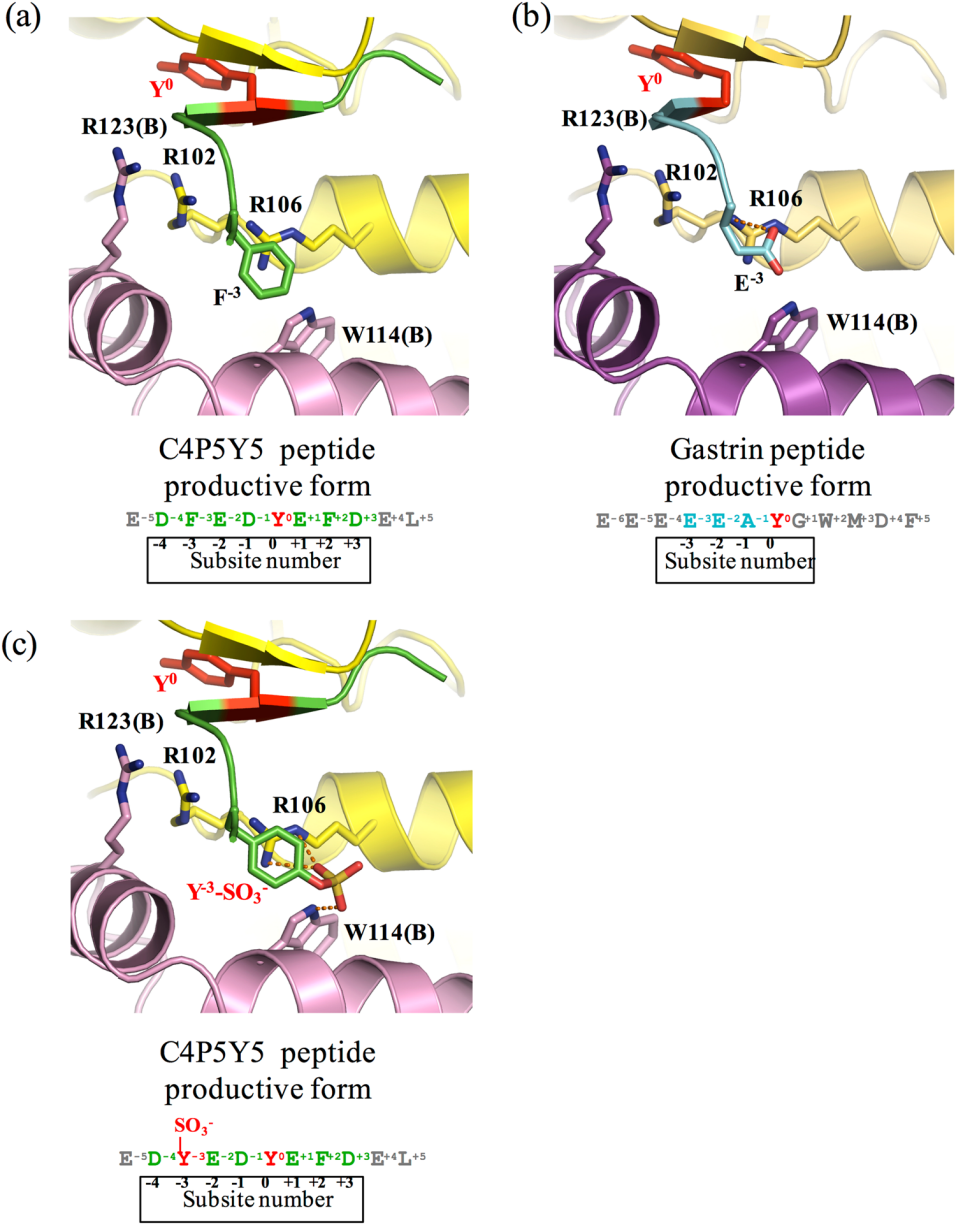
Recognition of the residue at the subsite −3 in substrate peptides. Interaction between human TPST1 and the residue at the −3 position in (**a**) C4P5Y5 and (**b**) the gastrin peptide productive form. (**c**) Interaction between human TPST1 and the sulfated tyrosine residue at the subsite −3 in the C4P5Y5 model peptide. The sulfotyrosine residue at the subsite −3 in C4P5Y5 is recognized by human TPST1 through electrostatic and hydrophobic interactions. Hydrogen bonds are depicted as orange dotted lines.

In the human TPST1-PAP-C4P5Y5 complex, eight residues (D^−4^, F^−3^, E^−2^, D^−1^, Y°, E^+1^, F^+2^, D^+3^) out of the eleven residues of C4P5Y5 were defined in the electron density map, suggesting that human TPST1 binds strongly to C4P5Y5. The human TPST1 recognizes C4P5Y5 in a manner similar to that of human TPST2 (Supplementary Fig. S7). Here, subsite numbers −6 to +6 for binding to each side chain of the substrate peptide are defined (Figs 3,4 and 5). The site at which SO_3_^−^ is accepted is defined as subsite 0.

In the human TPST1-PAP-gastrin peptide complex, the electron density of substrate peptide binding area could not be interpreted with any part and any one conformation of gastrin peptide. We could interpret the electron density with two binding patterns of gastrin peptide each having 0.5 occupancies (Fig. 3b,c, Supplementary Fig. S8). Though other possibility could not be excluded completely, this interpretation is thought to be reasonable based on peptide sequence and fit to the electron density. The relatively close average b-factor values (58 Å^2^ and 46 Å^2^) between refined peptide atoms and refined protein atoms (Table 2) also supports this interpretation. The two binding patterns; four residues (E^−3^, E^−2^, A^−1^, Y°) and (E^−4^, E^−3^, E^−2^, A^−1^) out of the twelve gastrin peptide residues were defined. We could model these two patterns with 0.5 occupancies each for all four active sites in asymmetric unit. In one binding pattern, four ordered residues (E^−3^, E^−2^, A^−1^, Y°) in the gastrin peptide are thought to constitute a productive form based on the position of Y° (Fig. 3b,e), whereas in the other binding pattern, four ordered residues (E^−4^, E^−3^, E^−2^, A^−1^) in the gastrin peptide are thought to constitute a nonproductive form (Fig. 3c,f).

## Discussion

In human, two isoforms of TPST1 and human TPST2 mediate the tyrosine sulfation reaction. However, the detailed catalytic mechanism of human TPST1 has remained elusive. In this study, we determined the crystal structure of the human TPST1-PAP-C4P5Y5 and human TPST1-PAP-gastrin peptide complexes. Basically, the structural similarity of human TPST1 and human TPST2 indicated that human TPSTs share the same catalytic mechanism. Furthermore, we have provided additional insights into the mechanism describing how human TPSTs recognize various substrate proteins as described below.

The structures of two substrate peptides indicate that all substrate peptides adopted an L-shaped conformation, consistent with a previous report of human TPST2^26^. This suggests that the vicinity of Y° on substrate proteins should probably adopt an intrinsically flexible conformation to facilitate active site binding to human TPST1.

Though central part of substrate binding region is well conserved between human TPST1 and TPST2, further parts are not (Supplementaly Fig. S7a and b). These variations may explain the slight difference in substrate specificity between TPST1 and TPST2.

The total calculated interaction energy between human TPST1 with C4P5Y5 or the gastrin peptide based on the crystal structures was estimated to be −292 kcal/mol and −86 to −87 kcal/mol, respectively (Fig. 4a and b). These results are consistent with kinetic constants for the two peptides. Consequently, the calculated interaction energy of Y° and D^−1^ in C4P5Y5 was much lower than those of residues at other positions. Y° and D^−1^ therefore interact with human TPST1 more strongly than residues located at other positions. In particular, the calculated interaction energy of D^−1^ was the lowest when compared with residues at other positions of the substrate peptide and was much lower than that of A^−1^ in the gastrin peptide. The calculated interaction energies of residues furthest from the −1 and 0 positions were high. These results suggest that human TPST1 recognizes strongly Y° and the acidic residue at the −1 position. The contribution of these two residues to the binding to human TPST1 suggests that other residues might contribute much less to binding. As a result, this indicates that these two residues are key factors that define the mechanism of how human TPSTs recognize various substrates.

In the human TPST1-PAP-gastrin peptide complex, the gastrin peptide forms dual binding patterns. This is because the total interaction energy of the productive form is near equal to that of the nonproductive form (Fig. 4b,c) due to the equality of the total interaction energy at positions −1 and 0. The dual binding patterns may give rise to distinct binding of the substrate protein to human TPST1. Such dual binding patterns are consistent with the *K*_m_ values of the substrate peptides. Dual binding patterns and the lack of acidic residues at the −1 and +1 positions in the substrate peptide cause disorder to residues at the −4, +1, +2, +3 and +4 positions in the gastrin peptide and thus a high *K*_m_ value. Interaction of the gastrin peptide with TPST1, therefore, is quite different from that of C4P5Y5 because of the different amino acid sequences.

It is noted that the two binding patterns, while sharing similarity for residues at the −2 and −3 positions (Supplemental Fig. S9), differ for residues at the −1 and 0 positions (Fig. 3e,f). In the productive human TPST1-PAP-gastrin peptide complex, unlike the case with C4P5Y5, Y° and A^−1^ form some interactions with the active site of human TPST1 (Fig. 3d,e). The backbone of Y^0^ interacts only with the backbone of T199, whereas backbone of A^−1^ interacts only with the hydroxyl group of T199. These results indicate that the acidic residue at the −1 position is required for strong binding to TPSTs. In contrast, in the nonproductive human TPST1-PAP-gastrin peptide complex, unlike in the case with C4P5Y5, A^−1^ and E^−2^ at the 0 and −1 position also form some interactions with the active site of TPST1. The backbone of A^−1^ interacts only with the backbone of T199, whereas, consistent with that of D^−1^ in C4P5Y5, the side chain of E^−2^ is recognized by the side chains of R123 and R285.

The backbone of E^−2^ interacts with the side chain of R106 (Supplemental Fig. S9). The acidic residues (E^−4^, E^−3^ and E^−2^) of the gastrin peptide form electrostatic interactions with the active site of human TPST1. Specifically, E^−3^ of the gastrin peptide interacts with R102, R106 and R123 of human TPST1 through electrostatic interactions, whereas F^−3^ of the C4P5Y5 peptide interacts with W114 of human TPST1 through a hydrophobic interaction. Collectively, these results suggest that TPSTs recognize substrates through multiple interaction modes and thus are capable of recognizing a wide variety of protein substrates.

The residue at the −3 position of the substrate forms multiple interactions with human TPST1. F^−3^ in C4P5Y5 interacts with W114 of human TPST1 through a hydrophobic interaction (Fig. 5a). However, E^−3^ in the gastrin peptide interacts with R102, R106 and R123 of TPST1 through electrostatic interactions (Fig. 5b). There are hydrophobic and hydrophilic regions on the surface of TPSTs in almost all substrate-binding areas. Therefore, these results suggest that TPSTs may recognize different protein substrates based on multiple interaction modes and that TPSTs can recognize various substrates at multiple positions in a protein substrate.

In C4P5Y5, two Tyr residues (Y738 and Y743) in the original sequence have been mutated to Phe and only one Tyr (Y741) residue is present in the peptide. As described above, the active site of human TPST1 recognizes the residue at the −3 position in the substrate through electrostatic and hydrophobic interactions. The results suggest that the Tyr residue and sulfated Tyr residue can then be recognized by TPSTs through both types of interactions at the −3 position (Fig. 5c). Taken together, following the sulfation of a tyrosine residue, TPST1 may recognize the sulfated tyrosine residue through multiple interactions and continue to sulfate tyrosine residues at other positions.

## Conclusions

Information derived from the two crystal structures of human TPST1, in combination with those obtained from enzymatic characterization and interaction energy calculations, has provided insights into how TPSTs recognize various substrate proteins with different efficiencies. The current study provides additional information of molecular mechanism of TPSTs in mediating post-translational tyrosine.

## Materials and Methods

### Cloning, expression, and purification of recombinant human TPST1

To circumvent potential complications associated with the crystallization of membrane proteins, we opted to overexpress the catalytic domain (Lys43–Glu370) of human TPST1 that lacks the N-terminal tail, the transmembrane domain and part of the stem region. cDNA encoding Lys43–Glu370 of human TPST1 was produced using total gene synthesis and cloned into pUC57 (GenScript, Piscataway, NJ). The human TPST1 cDNA was then subcloned into pET15b (Novagen, Madison, WI), which provides an N-terminal 6 × His tag and a thrombin cleavage site. The prepared expression vector construct (designated pET15b-human TPST1) was sequenced to confirm the reading frame and fidelity of the coding region using the Biostudy Support Division of FASMAC Co., Ltd. The expression and purification of the recombinant human TPST1 was based on the same protocol previously used for the preparation of recombinant human TPST2^26^. During the purification of recombinant human TPST1, a spontaneous proteolysis was detected. Since the molecular weight of the isolated protein was estimated to be 35 kDa, the purified domain was predicted to correspond to Lys43-Lys341 of human TPST1. pET15b harbouring the cDNA encoding Lys43-Lys341 of human TPST1 was subsequently prepared using the PCR-based Takara Prime STAR mutagenesis basal kit, with pET15b-human TPST1 (Lys43-Glu370) as the template. The new vector construct was transformed into Origami (DE3) cells (Novagen) carrying the pGro7 (Takara, Japan) plasmid encoding chaperonin proteins GroEL and GroES for the expression of recombinant human TPST1. After overnight induction at 30 °C, the cells were collected by centrifugation at 5,000 × *g* for 10 min, resuspended in lysis buffer (50 mM Tris-HCl, pH 8.0, 500 mM NaCl) and disrupted by sonication. The crude homogenate prepared was subjected to centrifugation at 20,000 × *g* for 30 min and the supernatant was loaded onto a Ni-NTA agarose gel column (Qiagen, Valencia, CA). The bound proteins were eluted from the column using the lysis buffer containing 500 mM imidazole. DTT (at the final concentration of 1 mM) was added to the eluted fraction and the recombinant human TPST1 was concentrated using a centrifugal filter (Amicon Ultra 10 K, Millipore), and further purified using a Superdex 200 16/60 column (GE Healthcare, Waukesha, WI). The eluted fractions containing recombinant human TPST1 were pooled. The sample was exchanged into 50 mM Tris-HCl (pH 7.0) and 200 mM NaCl buffer using a PD10 column (GE Healthcare). For crystallization of the human TPST1-PAP-gastrin peptide complex, the N-terminal His-tag of the recombinant human TPST1 was removed by overnight treatment with bovine thrombin (Sigma-Aldrich). Subsequently, thrombin was removed by benzamidine Sepharose 6B (GE Healthcare). The buffer was exchanged to 50 mM Tris-HCl (pH 7.0) and 200 mM NaCl using a PD10 column.

### Enzymatic assay

The sulfotransferase activity assay was performed as described previously with minor modifications^19^. The standard assay mixture (with a 20 µl final volume) consisted of 50 mM MES-NaOH, pH 6.0, 50 mM NaF, 20 mM MnCl_2_, 2 µg BSA, 0.5 µM [^35^S] PAPS, a substrate peptide (C4P5Y5 or the gastrin peptide) and 0.6 µg (for C4P5Y5) or 4.0 µg (for the gastrin peptide) human TPST1. The reaction proceeded at 30 °C for 15 min and was terminated by heating at 100 °C for 3 min. The precipitates formed were cleared by centrifugation and the supernatants collected were analysed for [^35^S] sulfated peptides by a thin-layer chromatography (TLC) procedure, with *n*-butanol/pyridine/formic acid/water (5:4:1:3; by volume) as the solvent system. Subsequently, the plates were air-dried and analysed using a Fluoro Image Analyzer (FLA-3000G).

### Crystallization and data collection

Human TPST1-PAP-C4P5Y5 crystals were prepared by crystallizing purified recombinant human TPST1 (2.5 mg/ml) together with 2 mM PAP and 1 mM C4P5Y5 using the sitting-drop vapor-diffusion method at 20 °C. Crystals were grown under a reservoir solution consisting of 0.2 M trimethylamine N-oxide dihydrate, 0.1 M Tris-HCl, pH 8.5, and 20% w/v polyethylene glycol monomethyl ether 2,000 for nine months. For data collection, a crystal was transferred to the cryo buffer (0.2 M trimethylamine N-oxide dihydrate, 0.1 M Tris-HCl, pH 8.5, 20% w/v polyethylene glycol monomethyl ether 2,000 and 30% glycerol). Thereafter, the preparation was flash-frozen using a cryo system (Rigaku). X-ray diffraction datasets were collected on the beamline BL38B1 at SPring-8, Hyogo, Japan. The diffraction data were processed using the program package HKL2000^29^. The crystal was found to belong to the space group *P*2_1_2_1_2. The unit cell parameters were *a* = 78.6 Å, *b* = 156.0 Å, and *c* = 48.9 Å. Data collection statistics are summarized in Table 2.

Human TPST1-PAP-gastrin peptide crystals were prepared by crystallizing purified recombinant human TPST1 (5.0 mg/ml) together with 2 mM PAP and 3 mM gastrin peptide using the sitting-drop vapor-diffusion method at 20 °C. Crystals were grown under a reservoir solution consisting of 0.2 M potassium sodium tartrate trihydrate and 19.5% w/v polyethylene glycol 3,350 for two months. Subsequently, the crystals were soaked for 1 d in a 350 nL solution containing 30 mM gastrin peptide. For data collection, a crystal was transferred to the cryo buffer (50 mM Tris-HCl, pH 7.0, 200 mM NaCl, 0.2 M potassium sodium tartrate trihydrate, and 19.5% w/v polyethylene glycol 3,350, 15% v/v glycerol). Thereafter, the preparation was flash-frozen using the cryo system. X-ray diffraction datasets were collected on the beamline BL38B1 at SPring-8, Hyogo, Japan. The diffraction data were processed using the program package HKL2000. The crystal was found to belong to the space group *P*1. The unit cell parameters were *a* = 51.7 Å, *b* = 93.3 Å and *c* = 93.0 Å. Data collection statistics are summarized in Table 2.

### Structural solution and refinement

The crystal structures of the human TPST1-PAP-C4P5Y5 and human TPST1-PAP-gastrin peptide complexes were determined by molecular replacement using Molrep^30^. For TPST1-PAP-C4P5Y5, the structure of the human TPST2-PAP-C4P5Y3 complex (Protein Data Bank accession entry 3AP1) was used as the search model. For the human TPST1-PAP-gastrin peptide complex, the structure of human TPST1-PAP-C4P5Y5 was used as the search model. The two structures were manually modified using the Coot program^31^. Several iterative rounds of model building were performed in Coot and refined using Refmac5^32^. Refinement statistics are summarized in Table 2. In the human TPST1-PAP-C4P5Y5 structure, Tyr65-Pro338 and Ala64-Gly336 were refined for protomer-1A and 1B, respectively.

In the human TPST1-PAP-gastrin peptide structure, Ala64-Pro338, Ala64-Pro338, Ala64-Gly336, and Tyr64-Pro338 were refined for protomer-1A, 1B, 1 C, and 1D, respectively. The quality of the structures was assessed using PROCHECK^33^. The atomic coordinates and structural parameters of the human TPST1-PAP-C4P5Y5 complex and human TPST1-PAP-gastrin peptide complex have been deposited in the Protein Data Bank under accession codes 5WRI and 5WRJ, respectively.

### Interaction energy calculation between human TPST1 and substrate peptides

The interaction energy between human TPST1 and the substrate peptides was calculated using MOE (2014.03; Chemical Computing Group Inc., Montreal, Canada) and the two crystal structures. Before the calculation, all water molecules in the structure were removed, proton atoms were added to all proteins and energy minimization of the added hydrogen atoms was performed using MOE.

## Electronic supplementary material

Supplementary Information

## References

[1] Bettelheim FR (1954). Tyrosine-O-sulfate in a peptide from fibrinogen. J. Am. Chem. Soc..

[2] Moore KL (2003). The biology and enzymology of protein tyrosine O-sulfation. J. Biol. Chem..

[3] Matsuzaki Y, Ogawa-Ohnishi M, Mori A, Matsubayashi Y (2010). Secreted peptide signals required for maintenance of root stem cell niche in Arabidopsis. Science.

[4] Gregory H, Hardy PM, Jones DS, Kenner GW, Sheppard RC (1964). The antral hormone gastrin. structure of gastrin. Nature.

[5] Leyte A (1991). Sulfation of Tyr1680 of human blood coagulation factor VIII is essential for the interaction of factor VIII with von Willebrand factor. J. Biol. Chem..

[6] Pouyani T, Seed B (1995). PSGL-1 recognition of P-selectin is controlled by a tyrosine sulfation consensus at the PSGL-1 amino terminus. Cell.

[7] Ippel JH (2009). Structure of the tyrosine-sulfated C5a receptor N terminus in complex with chemotaxis inhibitory protein of Staphylococcus aureus. J. Biol. Chem..

[8] Choe H, Farzan M (2009). Tyrosine Sulfation of HIV-1 Coreceptors and Other Chemokine Receptors. Methods Enzymol..

[9] Veldkamp CT (2008). Structural basis of CXCR4 sulfotyrosine recognition by the chemokine SDF-1/CXCL12. Sci. Signal..

[10] Farzan M (1999). Tyrosine sulfation of the amino terminus of CCR5 facilitates HIV-1 entry. Cell.

[11] Cormier EG (2000). Specific interaction of CCR5 amino-terminal domain peptides containing sulfotyrosines with HIV-1 envelope glycoprotein gp120. Proc. Natl. Acad. Sci. USA.

[12] Huang C (2007). Structures of the CCR5 N terminus and of a tyrosine-sulfated antibody with HIV-1 gp120 and CD4. Science.

[13] Tan Q (2013). Structure of the CCR5 chemokine receptor-HIV entry inhibitor maraviroc complex. Science.

[14] Hortin GL, Farries TC, Graham JP, Atkinson JP (1989). Sulfation of tyrosine residues increases activity of the fourth component of complement. Proc. Natl. Acad. Sci. USA.

[15] Bundgaard JR, Vuust J, Rehfeld JF (1995). Tyrosine O-sulfation promotes proteolytic processing of progastrin. EMBO J..

[16] Niehrs C, Huttner WB (1990). Purification and characterization of tyrosylprotein sulfotransferase. EMBO J..

[17] Ouyang YB, Lane WS, Moore KL (1998). Tyrosylprotein sulfotransferase: purification and molecular cloning of an enzyme that catalyzes tyrosine O-sulfation, a common posttranslational modification of eukaryotic proteins. Proc. Natl. Acad. Sci. USA.

[18] Beisswanger R (1998). Existence of distinct tyrosylprotein sulfotransferase genes: molecular characterization of tyrosylprotein sulfotransferase-2. Proc. Natl. Acad. Sci. USA.

[19] Mishiro E, Sakakibara Y, Liu MC, Suiko M (2006). Differential enzymatic characteristics and tissue-specific expression of human TPST-1 and TPST-2. J. Biochem..

[20] Zhou W, Duckworth BP, Geraghty RJ (2014). Fluorescent peptide sensors for tyrosylprotein sulfotransferase activity. Anal. Biochem..

[21] Seibert C (2008). Sequential tyrosine sulfation of CXCR4 by tyrosylprotein sulfotransferases. Biochemistry.

[22] Seibert C, Cadene M, Sanfiz A, Chait BT, Sakmar TP (2002). Tyrosine sulfation of CCR5 N-terminal peptide by tyrosylprotein sulfotransferases 1 and 2 follows a discrete pattern and temporal sequence. Proc. Natl. Acad. Sci. USA.

[23] Danan LM, Yu Z, Hoffhines AJ, Moore KL, Leary JA (2008). Mass Spectrometric Kinetic Analysis of Human Tyrosylprotein Sulfotransferase-1 and -2. J. Am. Soc. Mass Spectrom..

[24] Yu Y, Hoffhines AJ, Moore KL, Leary JA (2007). Determination of the sites of tyrosine O-sulfation in peptides and proteins..

[25] Lin WH, Larsen K, Hortin GL, Roth JA (1992). Recognition of substrates by tyrosylprotein sulfotransferase. Determination of affinity by acidic amino acids near the target sites. J. Biol. Chem..

[26] Teramoto T (2013). Crystal structure of human tyrosylprotein sulfotransferase-2 reveals the mechanism of protein tyrosine sulfation reaction. Nat. Commun..

[27] Huang SY (2012). PredSulSite: Prediction of protein tyrosine sulfation sites with multiple features and analysis. Anal. Biochem..

[28] Hartmann-Fatu C (2015). Heterodimers of Tyrosylprotein sulfotransferases suggest existence of a higher organization level of transferases in the membrane of the trans-Golgi Apparatus. J. Mol. Biol..

[29] Otwinowski Z, Minor W (1997). Processing of X-ray diffraction data collected in oscillation mode. Methods in Enzymology.

[30] Vagin A, Teplyakov A (2000). An approach to multi-copy search in molecular replacement. Acta Crystallogr. Sect. D Biol. Crystallogr..

[31] Emsley P, Cowtan K (2004). Coot: Model-building tools for molecular graphics. Acta Crystallogr. Sect. D Biol. Crystallogr..

[32] Murshudov GN, Vagin AA, Dodson EJ (1997). Refinement of macromolecular structures by the maximum-likelihood method. Acta Crystallogr. D. Biol. Crystallogr..

[33] Vaguine AA, Richelle J, Wodak SJ (1999). SFCHECK: a unified set of procedures for evaluating the quality of macromolecular structure-factor data and their agreement with the atomic model. Acta Crystallogr. D. Biol. Crystallogr..

