# Supplementary material for: Structural basis for the broad substrate specificity of the human tyrosylprotein sulfotransferase-1
Source: Sci Rep. 2017 Aug 18;7:8776. doi: 10.1038/s41598-017-07141-8 (PMC5562738; doi:10.1038/s41598-017-07141-8)
Supplement: Supplementary file 1 — Supplementary Information [file 41598_2017_7141_MOESM1_ESM.pdf]

Supplementary Information

Structural basis for the broad substrate specificity of the human tyrosylprotein sulfotransferase-1

Shinnosuke Tanaka[1], Toshiaki Nishiyori[1], Hidetaka Kojo[1], Reo Otsubo[2], Moe Tsuruta[1], Katsuhisa Kurogi[3], Ming-Cheh Liu[4], Masahito Suiko[3], Yoichi Sakakibara[3] & Yoshimitsu Kakuta[1,2,*]

[1]Department of Bioscience and Biotechnology, Faculty of Agriculture, Kyushu University, Hakozaki 6-10-1, Fukuoka 812-8581, Japan

[2]Graduate School of Systems Life Sciences, Kyushu University, Hakozaki 6-10-1, Fukuoka 812-8581, Japan

[3]Food Research Branch, Department of Biochemistry and Applied Biosciences, Faculty of Agriculture, University of Miyazaki, Miyazaki 889-2192, Japan

[4]Department of Pharmacology, College of Pharmacy, University of Toledo Health Science Campus, Toledo, Ohio 43614, USA.

*To whom correspondence should be addressed. Phone: +81-92-642-7159;
Fax: +81-92-642-7159; e-mail: kakuta@agr.kyushu-u.ac.jp

Number of supplementary figure, 9.

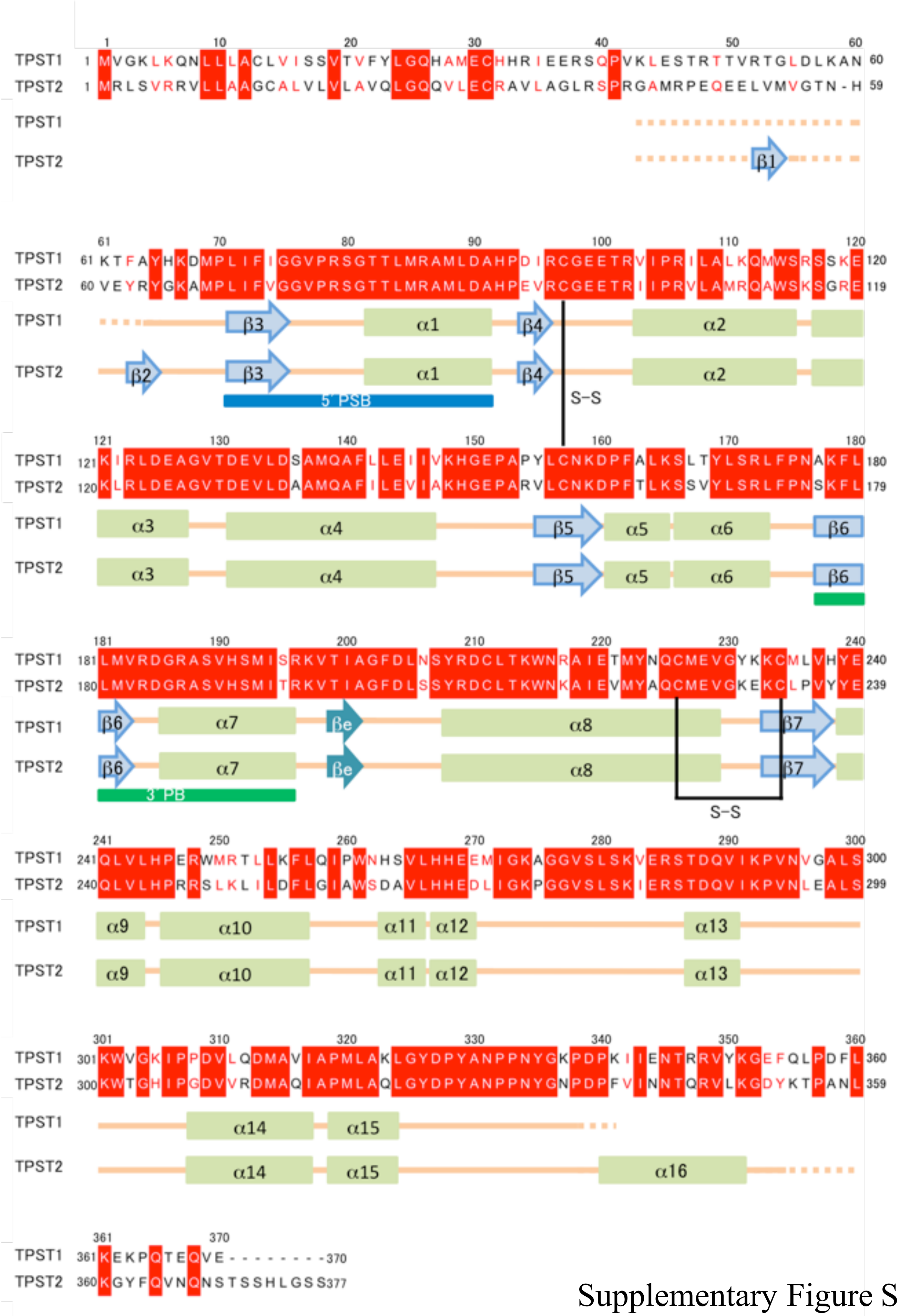

Supplementary Figure S1

Supplementary Figure S1. Sequence alignment of the secondary structures of human TPST1 and human TPST2. Region for crystallization of human TPST1 is Lys43–Glu341. The annotated secondary structures of human TPSTs are indicated below the alignment (arrows: β-strands, boxes: α-helices). β-strands paired with substrate peptide (C4P5Y3 and C4P5Y5) are indicated as βe. Disordered regions are indicated by dashed-lines. 5'PSB motif and 3'PB motif are shown by blue and green bars, respectively. The identical amino acids in the human TPSTs are boxed and coloured white. The homologous amino acids in the human TPSTs are coloured red. Two disulfide bonds (Cys97–Cys157 and Cys226-Cys234) revealed by the crystal structure of human TPST1-PAP-C4P5Y5 are shown in the figure. The only free Cys (Cys211) is buried in the interior of the protein molecule and is not exposed to solvent. This observation indicates that it is unlikely that the dimerization of the native enzyme could be stabilized by disulfide bonding between Cys211.

Supplementary Figure S1

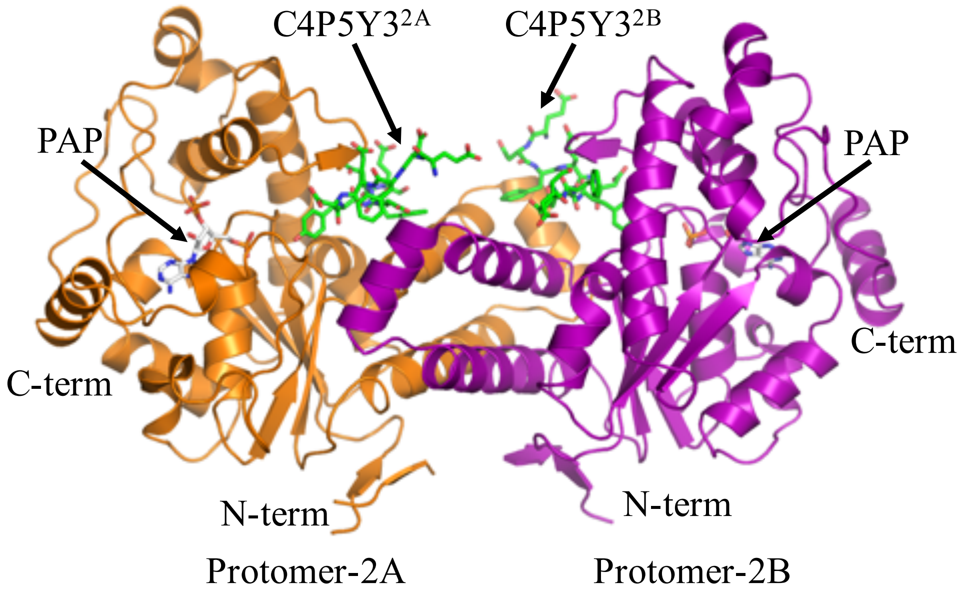

Supplementary Figure S2. Ribbon diagram of the structure of human TPST2-PAP-C4P5Y3 [19]. Protomer-2A and protomer-2B are orange and deep purple, respectively. C4P5Y3s are green. PAP is white. The dimeric complex has two active sites and binds two C4P5Y3s. To distinguish between two bound C4P5Y3s in human TPST2-PAP-C4P5Y3, the peptide sulfated by protomer-2A and protomer-2B are termed C4P5Y3^{2A} and C4P5Y3^{2B}, respectively.

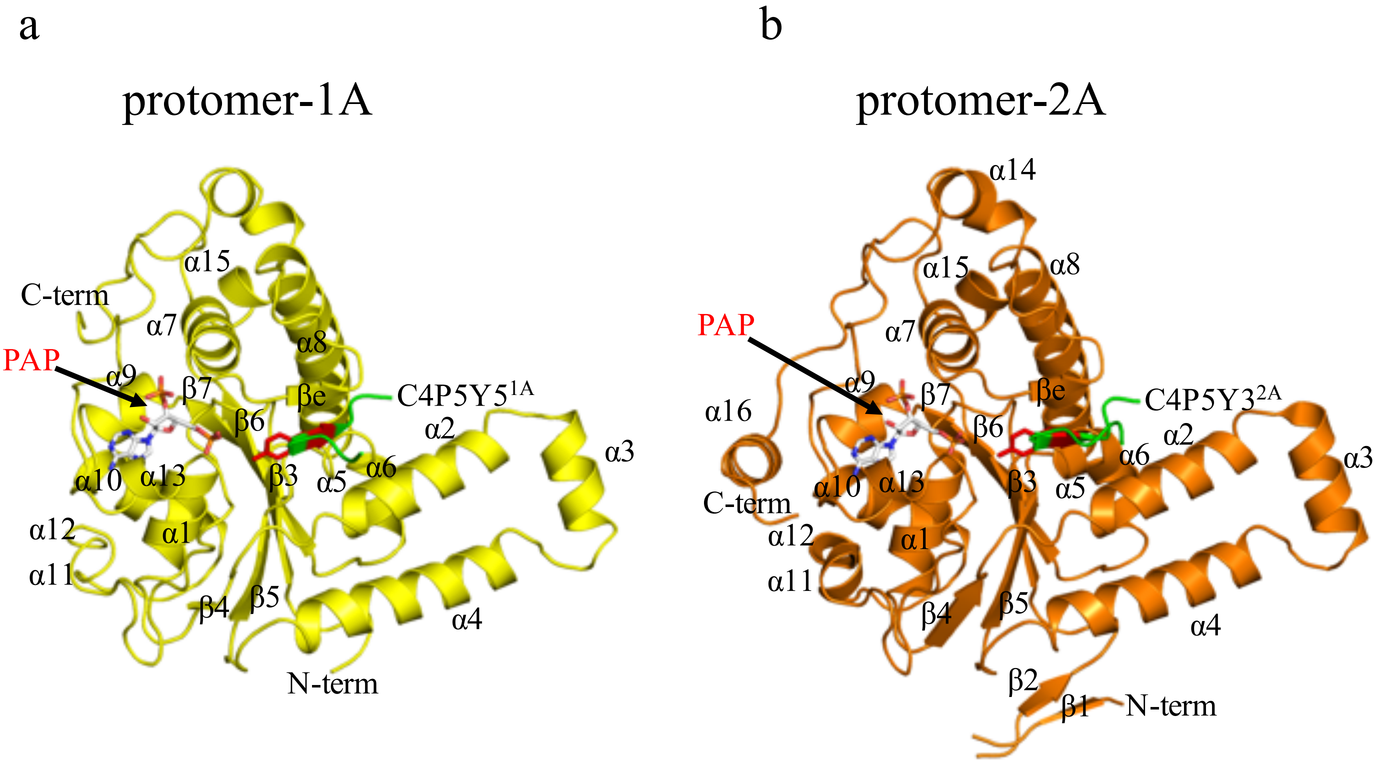

Supplementary Figure S3. Comparison of the human TPST1 structure with the human TPST2 structure. (**a-b**) Protomer ribbon diagrams of the human TPST1(yellow)-PAP(white)-C4P5Y5(green) and human TPST2(orange)-PAP(white)-C4P5Y3(green). (**a**) Crystal structure of protomer-1A of human TPST1-PAP-C4P5Y5. (**b**) Crystal structure of protomer-2A of human TPST2-PAP-C4P5Y3[19]. The human TPST1 catalytic domain comprises a single α/β motif with a five-stranded parallel β-sheet, flanked on both sides by α helices, and is consistent with the human TPST2 structure.

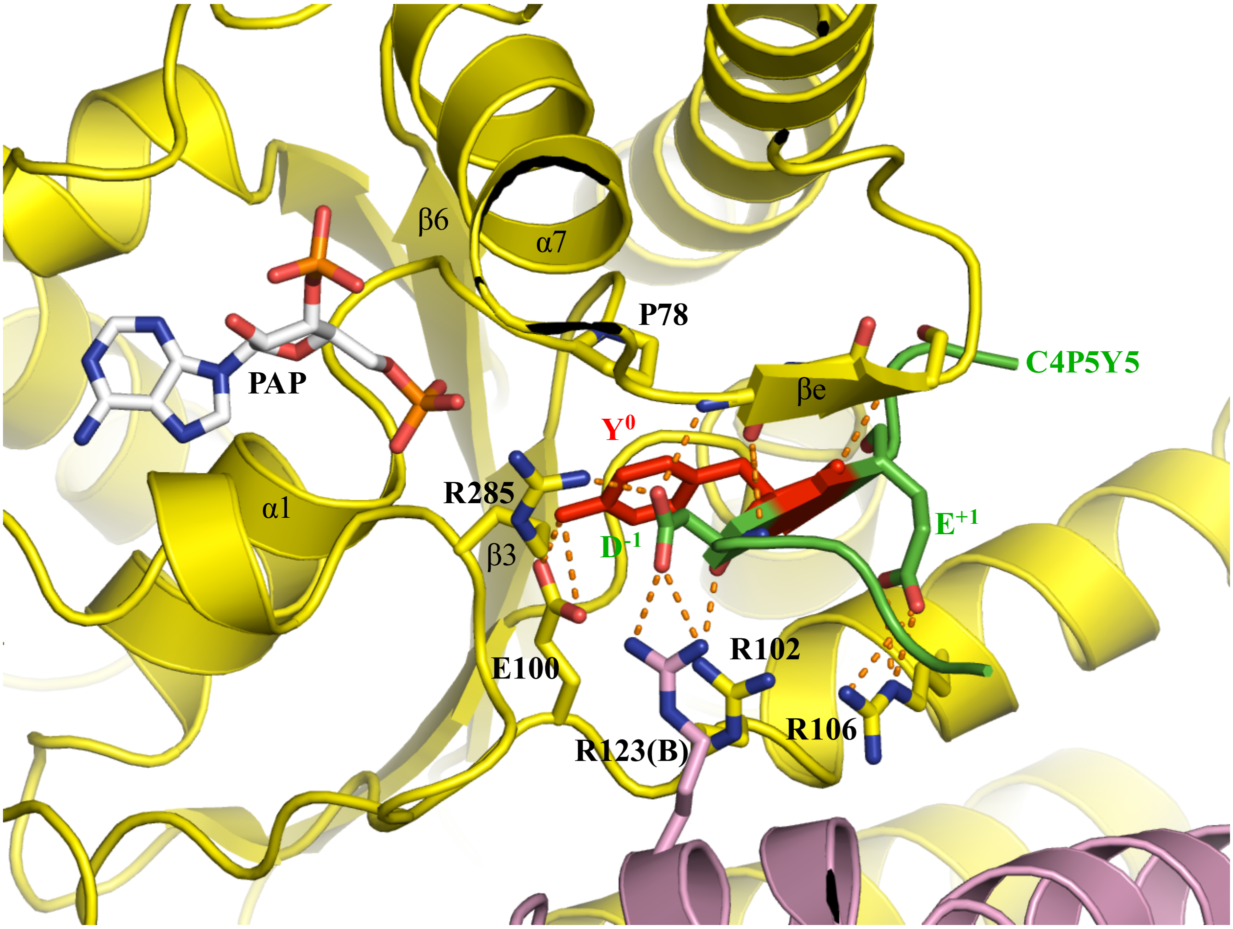

Supplementary Figure S4. Close-up views of the active site of human TPST1 in human TPST1-PAP-C4P5Y5. Hydrogen bonds are depicted as orange dotted lines. The identical interactions between two active sites in the dimer are shown. PAP is shown in stick model and coloured white. Central to this structural motif is the 5'-phosphosulfate-binding (5'-PSB) motif (76-GVPRSGTTL-84), contained within a strand-loop-helix consisting of β3 and α1. This loop forms extensive interactions with the 5'-phosphate of PAP. Moreover, β6 and α7 are also key elements that include the 3'-phosphate-binding (3'PB) motif of PAP. The 5'-PSB and 3'-PB motifs are conserved among all members of the sulfotransferase family.

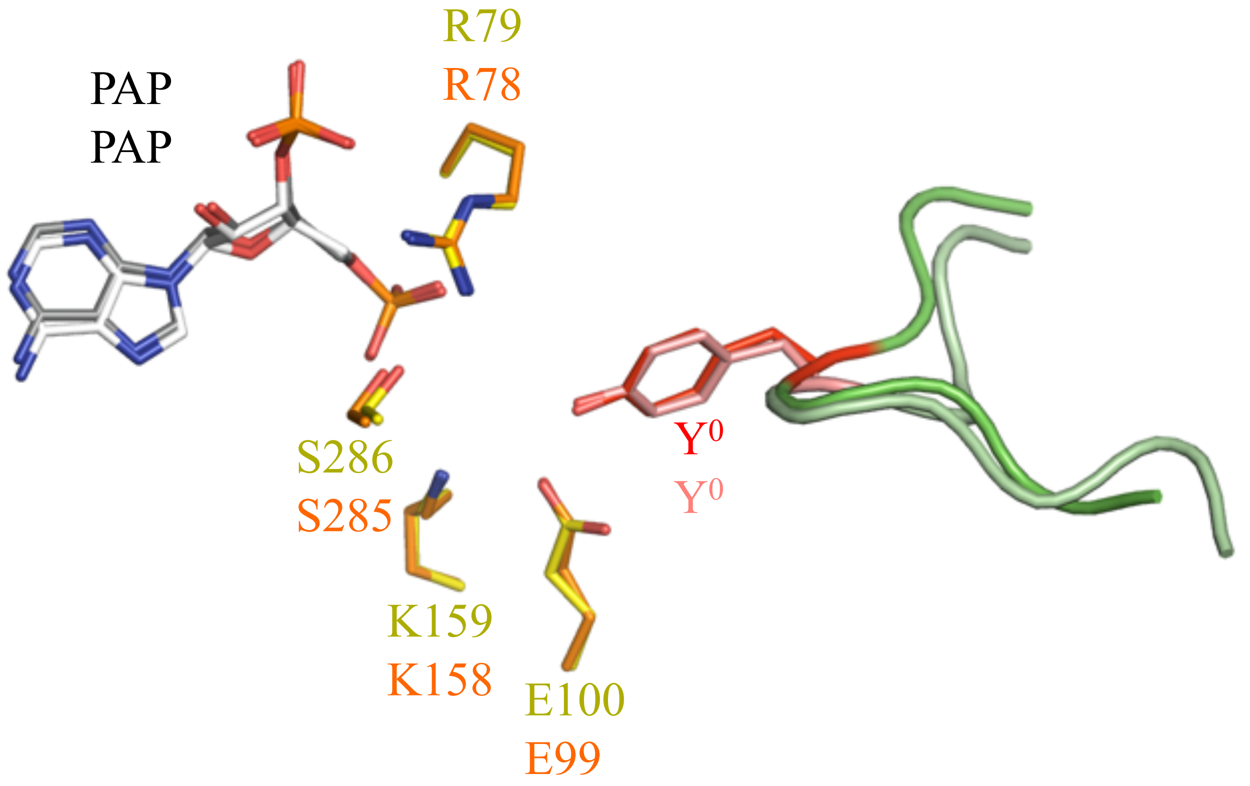

Supplementary Figure S5. Superposition of catalytic residues of human TPST1 and human TPST2. The catalytic residues of human TPST1 and human TPST2 are yellow and orange, respectively. Acceptor tyrosine residues of human TPST1 and human TPST2 are red and pink, respectively. C4P5Y5 in human TPST1-PAP-C4P5Y5 and C4P5Y3 in human TPST2-PAP-C4P5Y3 are green and pale green, respectively. PAP molecules in human TPST1-PAP-C4P5Y5 and human TPST2-PAP-C4P5Y3 are shown in a stick model and coloured gray and white.

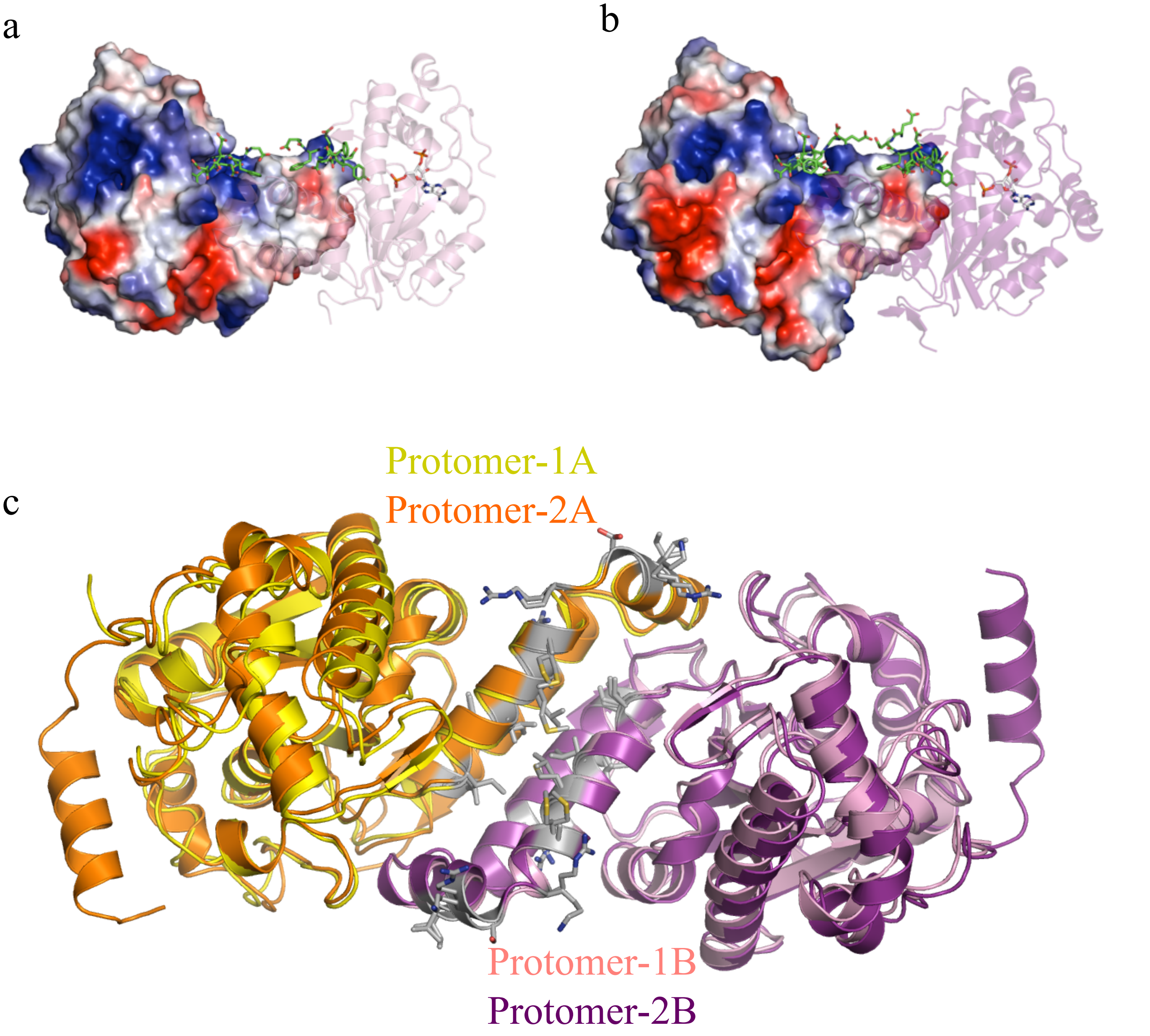

Supplementary Figure S6. Dimer interface of human TPST1 and human TPST2. (a-c) Dimer interfaces of human TPST1 and human TPST2 by surface representation. Positively charged surface regions are blue, whereas negatively charged surface regions are red. (a) Protomer-1A is shown in charged surface representation, whereas protomer-1B is shown in ribbon model and coloured pink. (b) Protomer-2A is shown in charged surface representation, whereas protomer-2B is shown in ribbon model and coloured purple. (c) Superposition of human TPST1 and human TPST2. The structure of human TPST1 and human TPST2 are shown in ribbon model. Protomer-1A and protomer-1B are yellow and pink, respectively. Protomer-2A and protomer-2B are orange and deep purple, respectively. Not conserved residues in dimer interface (α2, α3 and α4) are shown in stick model and colored grey.

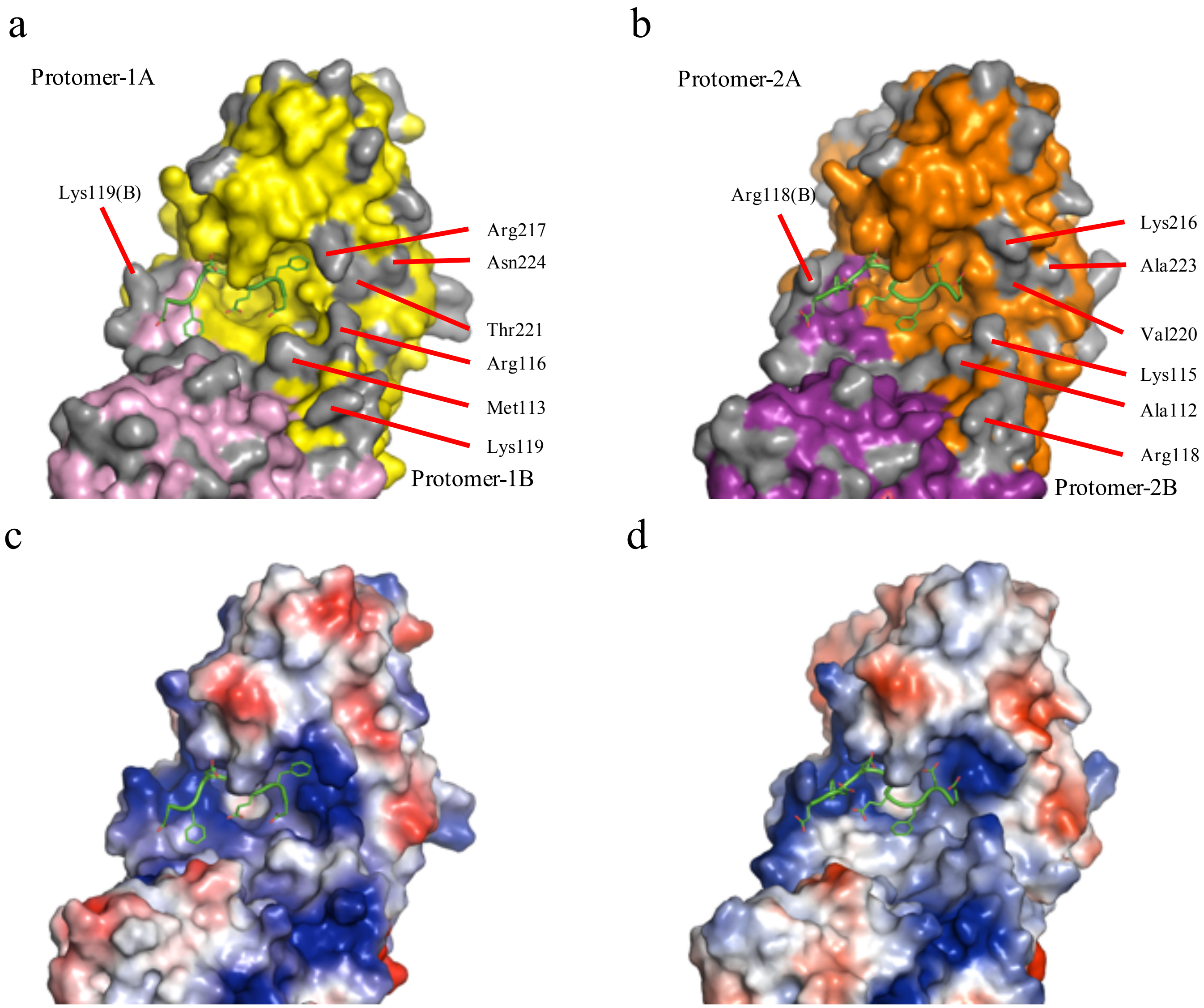

Supplementary Figure S7. Conservation surface representation and charged surface representation of human TPSTs. (a-b) Conservation surface representations of human TPST1-PAP-C4P5Y5 and human TPST2-PAP-C4P5Y3. Grey surfaces signify the not conserved area between human TPST1 and human TPST2. (**a**) Surface representation of human TPST1-PAP-C4P5Y5. (**b**) Surface representation of human TPST2-PAP-C4P5Y3. (**c-d**) Charged surface representation. Positively charged surface regions are blue, whereas negatively charged surface regions are red. (**c**) Charged surface representation of human TPST1-PAP-C4P5Y5. (**d**) Charged surface representation of human TPST2-PAP-C4P5Y3.

Supplementary Figure S7

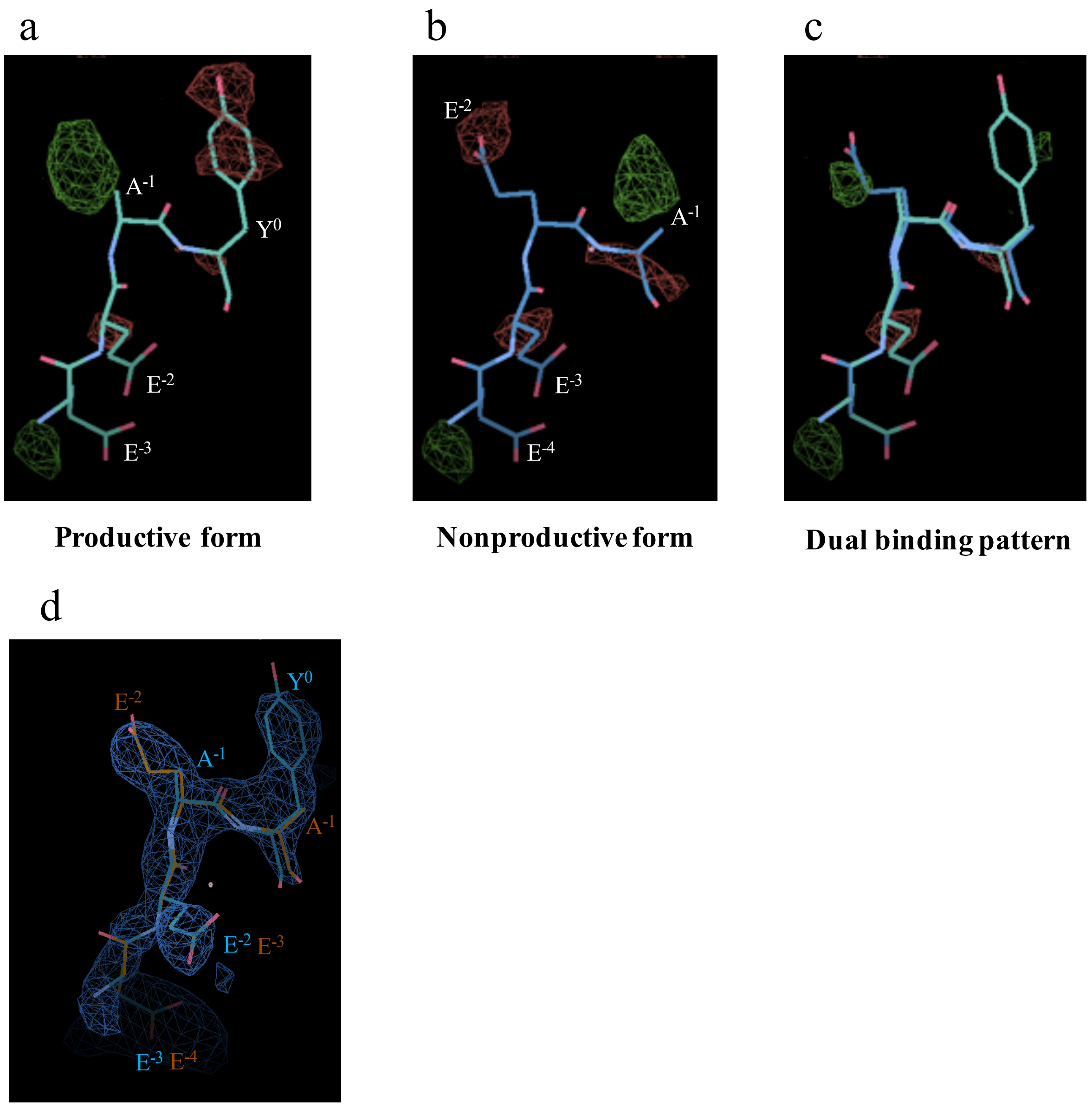

Dual binding pattern

Supplementary Figure S8. Dual binding pattern of the gastrin peptide. (**a-c**) Difference (Fo-Fc) electron density maps coloured at 2.86 σ with green as positive and red as negative. Residues in the substrate peptides are shown as stick models. All atoms of residues are shown in red and blue for oxygen and nitrogen, respectively. (**a**) The productive form of the gastrin peptide is shown as a stick model and coloured turquoise. (b) The nonproductive complex of the gastrin peptide is shown as a stick model and coloured blue. (**c**) The productive complex and nonproductive complex of the gastrin peptide are shown as a stick model and coloured turquoise and blue, respectively. (d) Polder map are contoured at the 4.00 σ as blue. The productive complex and nonproductive complex of the gastrin peptide are shown as a stick model and coloured turquoise and brown, respectively.

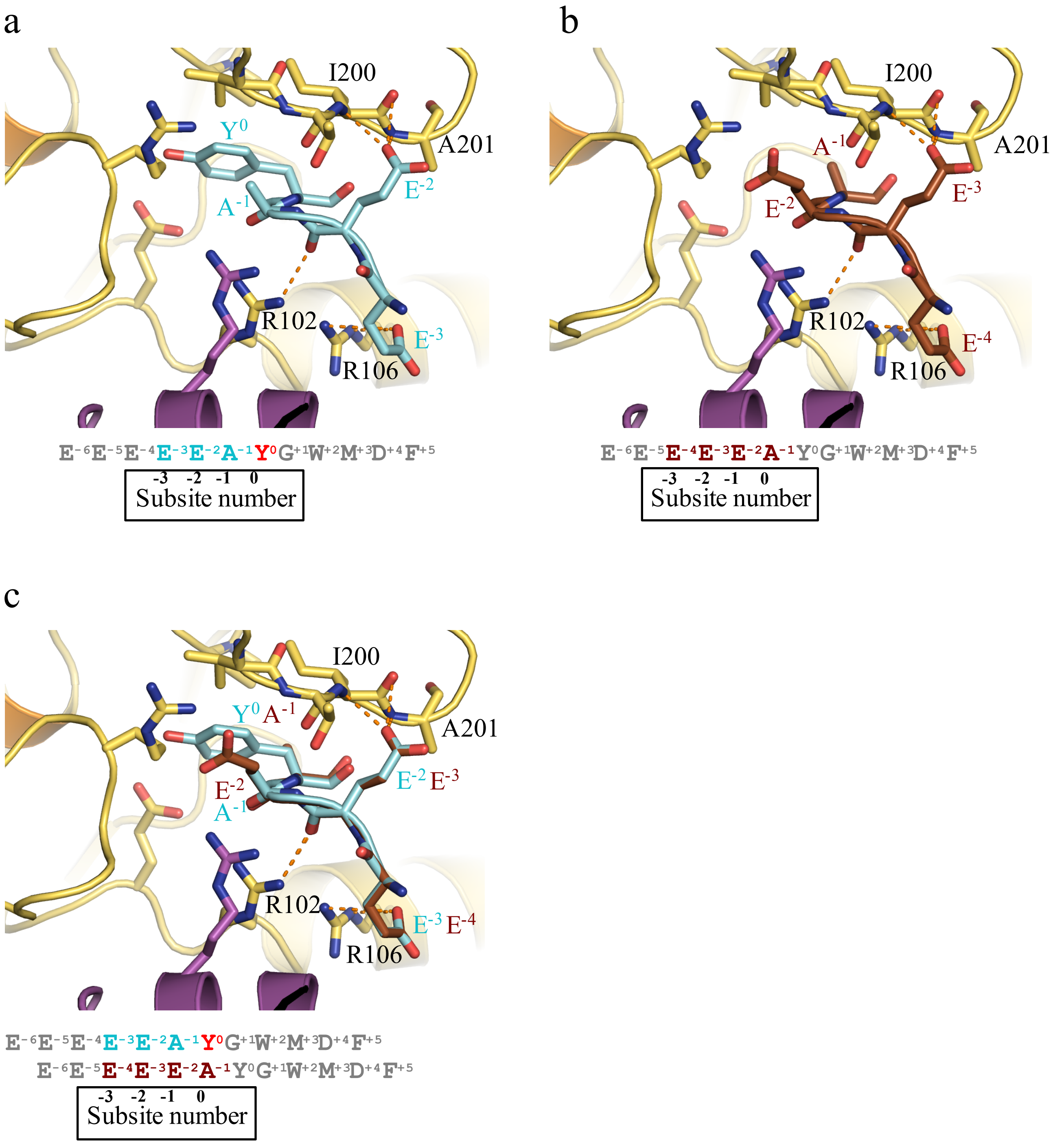

Supplementary Figure S9. Gastrin peptides in the active site of human TPST1 at subsite -2 and subsite -3.
(a) The productive form of the gastrin peptide is shown as a stick model and coloured turquoise.
(b) The nonproductive complex of the gastrin peptide is shown as a stick model and coloured brown.
(c) The productive complex and nonproductive complex of the gastrin peptide are shown as a stick model and coloured turquoise and brown, respectively. Hydrogen bonds are depicted as orange dotted lines. Amino acid sequences and subsite numbers are shown below.
